# We are what we eat: Implications of host plant suitability on sustainable production of silkworm pupae as novel ingredient with dietary and health benefits

**DOI:** 10.1371/journal.pone.0316290

**Published:** 2024-12-30

**Authors:** Marie N. Sangha, Didier K. Barwani, Cheseto Xavier, Linah Muhonja, Kelvin Moseti, Paul N. Karanja, Peter Kiiru, Isaac M. Osuga, Chrysantus M. Tanga

**Affiliations:** 1 International Centre of Insect Physiology and Ecology (*icipe*), Nairobi, Kenya; 2 Department of Animal Sciences, Jomo Kenyatta University of Agriculture and Technology, Nairobi, Kenya; 3 Department of Animal Production, Faculty of Agriculture and Environmental Sciences, Université de Kalemie, Kalemie, DR. Congo; 4 National Sericulture Research Centre, Kenya Agricultural & Livestock Research Organization, Nairobi, Kenya; 5 Department of Food Science and Technology, Jomo Kenyatta University of Agriculture and Technology, Nairobi, Kenya; Lusofona University of Humanities and Technologies: Universidade Lusofona de Humanidades e Tecnologias, PORTUGAL

## Abstract

Although, the impact of mulberry leaves on mortality of *Bombyx mori* [silkworm] and cocooning rates has been investigated, limited research has exploited the influence on the quality of *B*. *mori* pupae. This study evaluated the effects of four mulberry cultivars (Ichinose, Noi-EX-Thailand, Ex-Thika and Victory1) on the nutritional quality of *B*. *mori* pupae. The proximate composition, amino acids, vitamins, and minerals of different mulberry cultivars and silkworm pupae were analyzed using standard methods. Silkworms fed on Noi-EX-Thailand and Ex-Thika cultivars exhibited the highest crude protein (57.9%) and fat content (44.3%), respectively. Eight essential amino acids were identified in silkworm pupae, with histidine (6.02–7.12 μg/100mg) being the most abundant, followed by significant amounts of lysine (1.40–5.03 μg/100 mg), leucine (1.96–2.47μg/100 mg), and valine (0.89–2.03 μg/100 mg). Pupae raised on Noi-Ex-Thailand leaves had the highest total amino acid content (23.01 μg/100 mg). Potassium was the major mineral in pupae (505.62–665.30 mg/100 mg), with the highest levels observed in those fed on Noi-EX-Thailand. Vitamin C levels ranged from 28.84 to 61.88 mg/100 g, with pupae fed on Victory1 showing the highest concentration. There was a strong positive correlation between magnesium, crude protein and nitrogen-free extracts of mulberry leaves that mirrored the corresponding levels in silkworm pupae. These results underscore the importance of mulberry leaf composition in determining silkworm pupae quality, especially when considering their use as sustainable ingredient for the food, feed and pharmaceutical industry.

## 1. Introduction

The growing global population is leading to steady increase in the demand for food and animal protein, which in turn raises the costs of traditional feed ingredients such as soybean meal, fish meal, and corn. This situation highlights the urgent necessity to investigate and assess alternative protein sources for animal feed. Mulberry silkworm pupae (*Bombyx mori*), a by-product of the sericulture industry, offer a promising alternative. Silkworms, classified under the phylum Arthropoda, class Insecta, and order Lepidoptera, are primarily valued for their role in silk production [1]. The pupae, which make up approximately 60% of the dry weight of the cocoon, are the stage commonly used in food and feed applications [2, 3]. Numerous studies have demonstrated that silkworm pupae offer a high-quality protein source suitable for various applications, including animal feed, human dietary supplements, organic fertilizers, and uses in pharmacology and cosmetology [2, 4–6]. Beyond its essential role in sericulture and the textile industry, *Bombyx mori* is increasingly recognized as a valuable model organism for screening chemical compounds, providing insights that can enhance various applications [7].

The silkworm, a monophagous insect, relies exclusively on mulberry leaves during its larval stage, with the nutritional quality of these leaves influencing about 35–38% of its growth, pupal development, and cocoon production [8–11]. The health of silkworms and the quality of silk they produce are greatly influenced by both the quality and availability of mulberry leaves, with other environmental factors contributing to the overall effects [12].

The mulberry tree (*Morus spp*.), belonging to the Moraceae family, is extensively cultivated as the primary food source for silkworms because its nutrient-dense and palatable leaves are more suitable than other leafy vegetables such as spinach and amaranth [13]. Globally, thousands of mulberry varieties are grown, primarily classified into three species: white mulberry (*Morus alba*), red mulberry (*Morus rubra*), and black mulberry (*Morus nigra*), alongside numerous hybrids [14]. Native to China, white mulberry is widely grown for its leaves, essential to silk production, and its mildly sweet fruits, ranging from white to purple, which are consumed fresh or processed. Popular varieties include Ichinose, Victory1, Noi-EX-Thailand, and Thai mulberry [14–16]. Red mulberry, native to North America, is a hardy species prized for its juicy, reddish-black fruits, cherished for their natural sweetness and used in products like jams, pies, and wines [17, 18]. Black mulberry, originating from southwestern Asia, produces large, dark purple fruits with a rich, tart-sweet flavor [19], commonly used in desserts and preserves. While all three species thrive in temperate to subtropical climates, they exhibit varying preferences: white mulberry is highly adaptable, black mulberry thrives in well-drained soils, and red mulberry excels in diverse forest ecosystems [14–16]. Beyond their agricultural and culinary uses, mulberries are valued for their medicinal properties, attributed to bioactive compounds like flavonoids and anthocyanins, which provide antioxidants and anti-inflammatory benefits [17, 20].

Mulberry leaves have been identified as an excellent nutrient resource with high content of protein, carbohydrates, vitamins, microelements, and dietary fiber [5, 6, 21]. Some reports indicated that mulberry leaves had high content of bioactive compounds, including phenolic acids, flavonoids, alkaloids, and γ-aminobutyric acid [6, 21]. These compounds had been confirmed to involve in antioxidation [3, 9], decreasing glycemia, antihypertensive, preventing atherosclerosis, and anti-inflammation. Additionally, 1-deoxynojirimycin, which is known as one of the main bioactive compounds in mulberry leaves, is a potent inhibitor of α-glycosidases and has shown potential therapeutic effects on many diseases, particularly type II diabetes [5, 6, 21].

While studies have focused on how different mulberry leaf varieties affect silkworm mortality and cocooning rates, research on their impact on the nutritional quality of silkworm pupae as food or feed ingredients remains limited. This study addresses this gap by analyzing the nutrient composition of silkworm pupae—including amino acid profiles, mineral and vitamin content, and proximate composition—when reared on four most widely used and distinct mulberry cultivars: Ichinose, Noi-EX-Thailand, Ex-Thika, and Victory1. A quantitative experimental design with a factorial approach was used to evaluate how different mulberry cultivars affect the nutritional quality of silkworm pupae under controlled conditions. Key metrics included dietary and health benefits of the resulting pupae, facilitating a comprehensive comparison of host plant suitability. The study highlights the potential of optimizing mulberry cultivars to improve the nutritional value of silkworm pupae for sustainable feed, food and health applications.

## 2. Materials and methods

### 2.1 Study area

Mulberry tree leaves are the exclusive food for silkworms. Production of mulberry leaves on scientific lines is essential for organizing sericulture on sound economic lines. Silkworms were reared on established mulberry cultivars at the National Sericulture Research Centre (NSRC), under Kenya Agriculture and Livestock Research Organization (KALRO), in Murang’a County located in central Kenya (0°59’00" S, 37°04’00" E; 1548 m ASL). Murang’a County has a total area of 2,559 km^2^ and lies in the Eastern part extending into the lowland areas with a minimum of 914 m above sea level and 3,353m above sea level along the slopes of the Aberdare Forest in the Western boundary of the County. According to [22], due to favorable climatic conditions in Kenya, mulberry is mostly cultivated under rain fed conditions. A number of mulberry cultivars mostly imported have been under study since 1979 at the NSRC, whereby all the geographical and environmental conditions [land scape, altitude, soils, soil fertility, soil texture, pH of soil, soil temperature, soil moisture content, water factor, atmospheric moisture, rainfall, chemical properties and others] influencing effective cultivation of high yielding cultivars in Kenya has been reported by Tuigong et al. [22]. According to [23] the County experiences a bi-modal rainfall with short rains from October to December and long rains from March to May. The County is divided into three climatic zones according to altitude. The upper zone extends from 1800 m to 2220 m asl, with the upper part marking the edge of the Aberdare Forest. Annual rainfall at this zone averages 1800 to 2000 mm. The middle zone extends from 1400 to 1800 m asl, with annual rainfall ranging from 1400 to 1600 mm. The lower zone extends from 900 to 1400 m asl, with annual rainfall averaging below 900mm. Soil types in the County differ from one zone to another, with the upper zone being largely dominated by volcanic ash [24]. Soils in the middle zone are largely dominated by nitosols, which are well-structured, nutrient-rich clay soils. Soils in the lower zone are mainly dominated by deep, strongly leached poor clay soils of the ferrosol category [24].

### 2.2 Sample collection and preparation

For each mulberry cultivar (Ichinose, Noi-EX-Thailand, Ex-Thika, and Victory1), 21 plants were randomly selected from the orchard. Mature leaves (250–300 g) were harvested, washed, and air-dried at room temperature. Half were used immediately for feeding experiments, and the other half was stored at -20°C for nutritional analysis.

**Fig 1:**
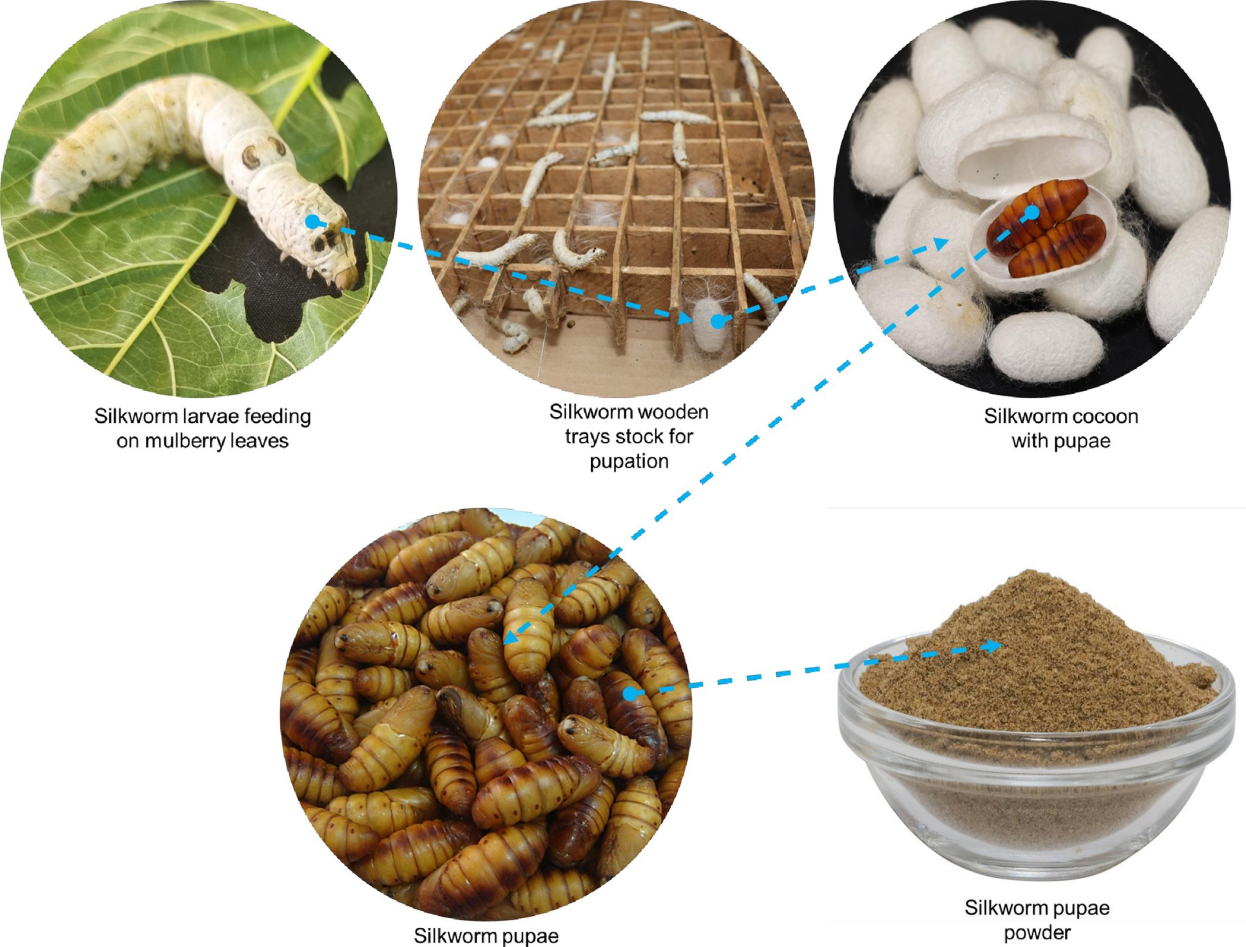
Illustration of the silkworm larval stage when fed on mulberry leaves and pupae powder product.

After 36 days of feeding the silkworm on different cultivars of mulberry leaves, 305 pupae from each feeding experiment were collected and weighed. The silkworm pupae and the respective mulberry leaves were then oven-dried at 60°C for 24 h [25]. The dried samples (plants and insects) were ground into powder (Fig 1), transferred into sample containers, and stored at -20°C until use.

### 2.3 Determination of nutritional composition

The nutritional analyses were performed at the International Centre of Insect Physiology and Ecology (*icipe*) (-1.221618, 36.896548) and at Jomo Kenyatta University of Agriculture and Technology (JKUAT) (1.0913° S, 37.0143° E). The silkworm pupae samples and their respective mulberry leaves were analyzed in triplicate for crude ash, crude protein, and ether extract (crude fat) using the methods by the Association of the Official Analytical Chemists (AOAC) [26]. Nitrogen content was determined using the Kjeldahl method, and the amount of crude protein was determined by multiplying the nitrogen value by 6.25. Carbohydrate content was determined by differences.

### 2.4 Determination of amino acids composition

The amino acid profiles of silkworm pupae and corresponding mulberry leaves were analyzed following acidic hydrolysis of the samples, with Liquid Chromatography Mass Spectrometry (LC-MS) used for detection, following the method outlined by [27].

### 2.5 Determination of minerals composition

The mineral composition of silkworm pupae silkworm and corresponding mulberry leaves was analyzed following the method in [28]. Each sample was tested in triplicate for minerals including iron, zinc, calcium, potassium, sodium, and magnesium, using dry-ashing and atomic absorption spectrophotometry (AAS, SHIMADZU -AA 7000). The procedure involved charring the samples, then incinerating it at a gradually increasing temperatures up to 250°C for 1 h, followed by 6 h at 600°C. The resulting ash was dissolved in 20 mL of 6N HNO3, heated at 80°C for 5 min, and then diluted to 100 mL with distilled deionized water. After filtering out insoluble matter, the solution analyzed with an AAS, using calibration curves generated with the mineral standards.

### 2.6 Determination of vitamin content

The water-soluble vitamins were analyzed following the method in [29]. Five grams of sample were homogenized in 20 mL of deionized for 1 min at medium speed. The mixture was centrifuged at 14 000 g for 10 min. Solid phase extraction was performed on the stationary phase (sep-pak C18 (500 mg) cartridges) which was activated and conditioned with 10 ml methanol and 10 ml of water adjusted to pH 4.2. The homogenized and centrifuged samples (10 ml) was loaded onto the cartridge, washed with 5 mL of water (pH 4.2), and then eluted with 10 mL of methanol at a flow rate of 1ml/min. The collected eluent was concentrated *in vacuo* and redissolved in 5 mL of 1 mol/ L KH2PO4 (pH 7): methanol (90:10), filtered using .0.45 μm syringe and analyzed (20 μL) by a Shimadzu 20 A HPLC system. The analysis was monitored using a photo-diode detector (Paris Waters 2996) at different wavelengths for specific vitamins: 234 nm (thiamine and pyridoxine), 266 nm (riboflavin), 282 nm (folic acid) and 261 nm (niacin). The mobile phase, a mixture of 0.1 M KH2PO4 (pH 7) and methanol (90:10), was filtered, degassed by sonication, and pumped at 0.7 mL/min flow rate through an ODS C-18 column at room temperature (25°C). Identification and quantification were based on matching retention times and peak areas with those of known standards.

### 2.7 Data analysis

Data on proximate analysis, amino acid profiles, minerals and vitamins composition of silkworm pupae grown on different mulberry leaf cultivars were subjected to Bartlett test to verify the homogeneity of variances then, one-way analysis of variance (ANOVA) in a completely randomized design was performed using R software for windows, version 4.3.0 (R core Team, 2023). Tukey’s multiple comparison test was used for the post-hoc analysis among the treatments at p<0.05. Pearson correlation was conducted to evaluate the relationship between different estimated nutrient composition of mulberry cultivars and silkworm pupae.

## 3. Results

### 3.1 Nutritional composition of silkworm pupae reared on different mulberry cultivars

The proximate composition of both the host plants and the pupae stages of the silkworm is presented in Table 1. Notable variations were observed in the parameters across the host plants, except for crude fat. Among the different host plants, silkworms fed on Noi-EX-Thailand exhibited the highest crude protein content in their pupae (57.9%). In contrast, the crude protein levels of pupae from silkworms raised on other host plants were similar.

**Table 1:** Proximate composition (% DM) of silkworm pupae reared on different mulberry leaf cultivars.

	Host plant type				*P*-values
Variable	Ichinose	Noi-EX-Thailand	Ex-Thika	Victory1	
Ash	18.03±0.31^b^	19.82±0.14^a^	14.59±0.31^c^	14.36±0.04^c^	<0.001
Crude fat	3.04±0.23	4.61±0.71	4.09±0.12	2.86±0.36	0.0532
Crude protein	19.37±0.11^b^	23.69±0.17^a^	19.31±0.06^b^	14.78±0.01^c^	<0.001
Total carbohydrates	59.56±0.29^c^	51.88±0.93^d^	62.00±0.31^b^	68.00±0.49^a^	<0.001
	Silkworm pupae				
Ash	7.72±1.43^b^	8.33±0.28^a^	4.59±0.76^c^	9.39±0.46^a^	0.0353
Crude fat	36.19±0.53^b^	32.98±0.20^c^	44.28±0.19^a^	33.68±0.35^c^	<0.001
Crude protein	51.12±0.35^b^	57.89±0.47^a^	47.91±1.37^b^	50.34±0.67^b^	<0.001
Total carbohydrates	4.96±1.75^b^	0.78±0.33^c^	3.20±1.34^ab^	6.57±0.79^a^	0.05

Means with different superscript letters in each row are significantly different at p < 0.05 (mean± Standard Error of Mean).DM: Dry matter basis.

### 3.2 Amino acid composition of silkworm pupae reared on different mulberry cultivars

The amino acid profiles of silkworm pupae reared on different mulberry cultivars are shown in Table 2. All the pupae reared on the different mulberry cultivars contained eight essential amino acids and five non-essential amino acids. Histidine was the most abundant amino acid, followed by notable levels of lysine and leucine.

**Table 2:** Amino acid composition (μg/100 mg) of silkworm pupae reared on different mulberry leaf cultivars.

Amino acids	Host plant type				P-value
	Ex-Thika	Ichinose	Victory1	Noi-EX-Thailand	
Gly	0.27±0.11ª	0.11 ±0.05ª	0.26 ±0.02ª	0.24±0.01ª	0.299
Lys	0.70±0.19ª	0.12 ±0.03ª	0.38 ±0.11 ª	0.40±0.02ª	0.219
His	1.34±0.03^b^	0.70 ±0.21^a^	1.15 ±0.15ab	0.63±0.03^a^	0.016
Arg	0.37±0.21^a^	0.09 ±0.03^a^	0.26±0.03^a^	0.39±0.14^a^	0.396
Glu	0.29±0.04^a^	0.38 ±0.04^a^	0.28±0.01^a^	0.41±0.02^a^	0.074
Pro	0.70 0.37^a^	0.36 ±0.50^a^	0.32±0.05^a^	1.31±0.30^a^	0.066
Val	1.48±0.52^a^	1.59 ±0.95^a^	0.79±0.20^a^	1.11±0.28^a^	0.754
Met	0.14±0.06^a^	0.14 ±0.02^a^	0.15±0.33^a^	0.32±0.11^a^	0.256
Tyr	0.58±0.21^a^	0.55±0.28^a^	0.57±0.15^a^	0.59±0.12^a^	0.999
Ile	0.86±0.10^a^	0.63±0.10^a^	0.53±0.04^a^	0.83±0.06^a^	0.074
Leu	1.15±0.08^a^	1.14±0.18^a^	1.17 ±0.11^a^	0.60±0.10^a^	0.089
Phe	1.72±0.36^a^	0.94±0.32^a^	1.64±0.01^a^	1.63±0.28^a^	0.203
Thr	0.34±0.02a	0.29±0.02a	0.26±0.00a	0.35±0.02a	0.059
	Silkworm pupae				
Gly	0.25±0.01^a^	0.22±0.01^a^	0.53±0.20^a^	0.35±0.03^a^	0.113
Lys	1.40±0.11c	2.59±0.40^a^	1.48±0.10c	5.03±0.13b	<0.001
His	7.12±1.47^a^	7.07±0.68^a^	6.25±1.23^a^	6.02±2.96^a^	0.959
Arg	0.48±0.16^a^	1.36±0.11^a^	1.11±0.49^a^	1.63±0.06^a^	0.077
Glu	0.21±0.02^a^	0.20±0.05^a^	0.24±0.02^a^	0.24±0.01^a^	0.767
Pro	0.23±0.01^a^	0.25±0.01^a^	0.29±0.02^a^	0.45±0.15^a^	0.282
Val	1.33±0.20^a^	0.89±0.13^a^	1.69±0.45^a^	2.03±0.46^a^	0.199
Met	0.51±0.15^a^	0.40±0.13^a^	0.76±0.05^a^	0.63±0.24^a^	0.491
Tyr	0.81±0.15^a^^b^	0.36±0.07^a^	1.13±0.12^b^	1.02±0.08^b^	<0.010
Ile	1.39±0.02bc	1.17±0.06^a^	1.20±0.03ac	1.49±0.04^b^	<0.010
Leu	1.96±0.06 ^a^	1.99±0.07^a^	2.07±0.04 ^a^	2.47±0.07^b^	<0.010
Phe	1.09±0.09^a^	1.69±0.19^a^	1.46±0.08^a^	1.36±0.21^a^	0.083
Thr	0.27±0.01^a^	0.31±0.02^a^	0.24±0.04^a^	0.29±0.02^a^	0.335

### 3.3 Mineral composition of silkworm pupae reared on different mulberry cultivars

The mineral composition of silkworm pupae reared on four different mulberry leaf cultivars is shown in Table 3. Potassium was the dominant mineral, ranging from 505.62 to 665.30 mg/100 g, with the highest level recorded in pupae fed on the Noi-EX-Thailand cultivar. No significant differences (P < 0.05) were found in the levels of calcium (Ca) and magnesium (Mg) among the silkworms reared on all cultivars.

**Table 3:** Mineral composition of silkworm pupae ((mg/100 g DM) reared on different mulberry cultivars mulberry cultivars.

Variable	Host plant type				P-values
	Ichinose	Noi-EX-Thailand	Ex-Thika	Victory1	
Calcium	2177.77±19.39	2853.72±7.37	2670.34±12.76	2246.12±9.91	0.530
Magnesium	294.56±2.06	309.54±2.72	285.33±4.11	284.80±3.09	0.760
Potassium	780.99±3.65	644.63±6.71	696.84±6.10	782.70±4.99	0.30
Sodium	84.66±2.95	41.23±1.88	54.29±3.98	87.60±2.91	0.250
Iron	19.37±1.33	14.83±1.38	16.13±1.20	13.04±0.23	0.380
Zinc	0.70±0.24	0.72±0.28	0.78±0.29	0.15±0.15	0.390
	Silkworm pupae				
Calcium	341.01±3.10	391.23±3.06	312.34±7.14	278.95±3.08	0.052
Magnesium	258.17±2.60	290.35±1.28	234.87±5.46	232.94±2.45	0.449
Potassium	565.05±3.45	665.30±1.90	505.62±8.80	518.17±3.92	0.405
Sodium	68.03±2.85	60.21±1.38	56.08±3.61	58.70±1.54	0.931
Iron	3.16±0.32	3.56±0.31	3.29±0.22	2.96±0.37	0.192
Zinc	0.38±0.18^c^	2.21±0.17^a^	1.06±0.46^b^	0.40±0.23^c^	< 0.001

Means with different superscript letters in each row are significantly different at p < 0.05 (mean± Standard Error of Mean).

### 3.4 Vitamin composition of silkworm pupae reared on different mulberry cultivars

The vitamin composition of silkworm pupae, reared on four different mulberry leaf cultivars, is summarized in Table 4. Significant differences (P < 0.05) were found in the levels of riboflavin, niacin, and folic acid among the silkworms reared on different mulberry leaves. The silkworm pupae demonstrated a rich vitamin content, with Vitamin C being the most abundant vitamin followed by thiamine and niacin. Notably, the highest concentrations of these vitamins were found in pupae fed on specific mulberry cultivars: Vitamin C was most abundant in those fed on Victory1, thiamine in those fed on Noi-EX-Thailand, and niacin in pupae fed on Ichinose.

**Table 4:** Vitamin composition (mg/100 g DM) of silkworm pupae reared on different mulberry cultivars.

Vitamin	Host plant type				P-values
	Ichinose	Noi-EX-Thailand	Ex-Thika	Vitory1	
Vitamin C	53.43±3.25^b^	163.17±2.22^a^	77.01±2.84^b^	198.28±2.41^a^	<0.001
Thiamine	4.10±0.33^b^	1.45±0.08^c^	7.89±0.60^a^	2.65±0.14^ab^	<0.001
Riboflavin	0.29±0.07^a^	0.28±0.01^a^	0.32±0.02^a^	0.27±0.01^a^	0.826
Niacin	2.41±0.52^a^	1.80±0.05^a^	3.84±0.32^b^	1.74±0.04^a^	<0.01
Pyridoxine	0.09±0.02^c^	1.01±0.04^a^	0.29±0.01^b^	0.40±0.01^b^	<0.001
Folic acid	0.11±0.03^b^	0.01±0.00^b^	0.24±0.03^a^	0.09±0.01^a^	<0.010
	Silkworm pupae				
Vitamin C	28.84±1.48^a^	42.60±2.18^a^	60.97±2.09^a^	61.88±2.68^a^	0.074
Thiamine	9.19±2.01^a^	12.12±1.19^a^	11.24±3.12^a^	8.96±1.55^a^	0.665
Riboflavin	0.33±0.05^a^	0.21±0.01^b^	0.16±0.00^ab^	0.19±0.01^ab^	0.050
Niacin	3.27±0.23^a^	2.58±0.08^a^	1.38±018^b^	2.90±.22^a^	<0.010
Pyridoxine	0.32±0.08^a^	0.41±0.03^a^	0.15±0.06^a^	0.30±0.07^a^	0.150
Folic acid	0.10±001^b^	0.12±0.00^a^	0.07±0.007^ab^	0.10±0.01^b^	0.050

Means with different superscript letters in each row are significantly different at p < 0.05 (mean± Standard Error of Mean).

### 3.5 Pearson’s correlation between the nutrient composition of silkworm pupae and the respective mulberry leaf cultivars

Table 5 illustrates the correlation between the nutrient composition of mulberry leaves and silkworm pupae. A negative correlation was observed between the ash content of the leaves and all other parameters, except for crude protein (CP) (r = 0.8214) and ash (0.1435) in the pupae. A strong positive correlation (p<0.01) between leaf ash content and pupae CP content suggests that mulberry species with higher ash content can also enhance the CP content of the pupae when these species serve as the primary feed source for the larvae. Additionally, leaf CP content showed a significant positive correlation with pupae CP (p<0.05) and a negative correlation with nitrogen-free extract (NFE) in the pupae (p<0.01). This indicates that pupae are likely to have higher CP levels when silkworm larvae are fed mulberry species with elevated CP content. Furthermore, the ether extract (EE) content of the leaves demonstrated a negative correlation (p<0.05) with the NFE of the pupae, while the NFE of the leaves exhibited a positive correlation (p<0.05) with the NFE of the pupae.

**Table 5:** Pearsons’s correlation coefficient for proximate components.

	Ash/pupae	CF/pupae	CP/pupae	EE/pupae	NFE/pupae
Ash/leaf	0.1435	-0.5781*	0.8214**	-0.5090	-0.5257
CF/leaf		-0.5149	0.2793	0.2105	-0.5700
CP/leaf			0.6788*	-0.0447	-0.8059**
EE/leaf				0.1590	-0.6324*
NFE/leaf					0.6847*

**Correlation is significant at the 0.01

* correlation is significant at the 0.05. CF, crude fibre; CP, crude protein. EE, ether extract; NFE, Nitrogen-free extract.

Table 6 presents the correlation coefficients among the eight essential amino acids found in mulberry leaves and silkworm pupae. A weak negative correlation was observed between the lysine content of the leaves and that of the pupae. However, the results indicate that pupae may exhibit higher levels of histidine, isoleucine, and threonine when the mulberry species used as feed contains greater lysine content. Although histidine showed a weak positive correlation (0.1258), it had positive correlations with methionine (0.0224) and isoleucine (0.0732) in the pupae. Valine displayed a very weak positive correlation (0.0015), but valine in the leaves showed weak positive correlations with methionine (0.0655) and phenylalanine (0.0002), as well as a moderate positive correlation with threonine (0.5702) in the pupae. The correlation coefficient for isoleucine (0.475) was moderately high, indicating a good relationship between these components in silkworm pupae and mulberry leaves. Leucine in the leaves also showed a positive correlation (p<0.05). A weak correlation (0.270) was recorded between threonine in mulberry leaves and silkworm pupae. In contrast, a weak negative correlation (-0.083) was found for phenylalanine in mulberry leaves and pupae.

**Table 6:** Pearsons’s correlation coefficient for essential amino acid components.

	Lys/pupae	His/pupae	Val/pupae	Met/pupae	Ile/pupae	Leu/pupae	Phe/pupae	Thr/pupae
Lys/leaf	-0.2503	0.2941	-0.3065	-0.0518	0.4015	-0.1936	-0.2677	0.0187
His/leaf		0.1258	-0.1658	0.0224	0.0732	-0.3672	-0.2660	-0.5133
Val/leaf			0.0015	0.0655	-0.1530	-0.1945	0.0002	0.5702
Met/leaf				-0.3316	0.2898	0.4508	-0.3250	0.2440
Ile/leaf					0.4752	0.0779	-0.3920	0.2464
Leu/leaf						0.5880*	-0.3164	0.1086
Phe/leaf							-0.0828	-0.2704
Thr/leaf								0.2700

^b^ correlation is significant at 0.05; Lys, Lysine; His, Histidine; Val, Valine; Met, Methionine; Ile, Isoleucine; Leu, Leucine; Phe, Phenylalanine; Thr, Threonine.

A positive correlation was observed between the protein content of silkworm pupae and the protein content of the mulberry leaves on which they were reared, as well as with their amino acid profile.

The correlation between the mineral composition of mulberry leaf cultivars and silkworm pupae is presented in Table 7. Calcium in mulberry leaves had a positive correlation with all silkworm pupae compounds except iron (r = -0.0539). The result showed that magnesium in mulberry leaves have high positive (p<0.05) correlation with magnesium in the pupae (r = 0.7063, P<0.05) and potassium in silkworm pupae (r = 0.7217, p<0.01). Zinc in mulberry leaves presented a positive correlation with all pupae components except iron. These results imply that when Ca and Zn from leaves increase, Fe from silkworm pupae would be low. Potassium leaf showed a weak negative correlation with potassium and sodium pupae.

**Table 7:** Pearsons’s correlation coefficient for mineral components.

	Ca/pupae	Mg/pupae	Zn/pupae	Fe/pupae	K/pupae	Na/pupae
Ca/leaf	0.2739	0.4479	0.5715	-0.0539	0.4374	0.0658
Mg/leaf		0.7063*	0.5112	0.0316	0.7217**	0.5155
Zn/leaf			0.4454	-0.0320	0.4248	0.3724
Fe/leaf				0.0257	-0.0230	-0.2819
K/leaf					-0.2446	-0.2167
Na/leaf						0.0173

** Correlation is significant at the 0.01

* correlation is significant at the 0.05; Ca, calcium. Mg, magnesium; Zn, zinc; Fe, iron; K, potassium; Na, sodium.

The Pearson’s correlation among vitamin components from mulberry leaves and silkworm pupae is presented in Table 8. The result showed that Vitamin C content of the leaves has a weak positive correlation with B3, B6 and B9 in silkworm pupae, excepts B1 and B2 in the pupae which were negative. Vitamin B1 in mulberry leaves has a negative correlation with all pupae components, except B9 in silkworm pupae. The high positive correlation (r = 0.7304, P<0.01) between vitamin B1 in silkworm leaf and vitamin B9 in silkworm pupae indicates that mulberry leaves are the primary source of folic acid (B9) for the growth and development of larvae and, therefore, for silkworm pupae. The result also revealed that vitamin B3 in mulberry leaf was negatively correlated (r = -0.6759, P<0.05) with vitamin B3 in pupae, while a similar result was also observed between vitamin B9 in mulberry leaf and vitamin B9 in silkworm pupae (r = -0.6882, P<0.05).

**Table 8:** Pearsons’s correlation coefficient for vitamin components.

	Vit C/pupae	B1/pupae	B2/pupae	B3/pupae	B6/pupae	B9/pupae
Vit C/leaf	0.3688	-0.0022	-0.4747	0.1391	0.2695	0.3170
B1/leaf		-0.0584	-0.2613	-0.6790*	-0.6391*	0.7304**
B2/leaf			-0.0765	-0.1441	0.3219	0.0626
B3/leaf				-0.6759*	-0.3360	-0.5437
B6/leaf					0.4412	0.5706
B9/leaf						-0.6882*

a Correlation is significant at the 0.01; b correlation is significant at the 0.05; Vit C, B1, B2, B3, B6, B9.

## 4. Discussion

The proximate composition of silkworm pupae was significantly influenced by the mulberry variety on which the larvae were reared. All mulberry cultivars exhibited comparable ash content, with the Noi-EX-Thailand cultivar exhibiting the highest level at 19.82%. This elevated ash content in the mulberry leaves was reflected in the pupae, which retained ash contents of 8.33% for Noi-EX-Thailand and 9.36% for Victory1. These results indicate that silkworms fed on these varieties retained appreciable amounts of minerals, as leaves with higher mineral content tend to enhance ash retention in the pupae. This trend aligns with the findings for both the Victory1 and Noi-EX-Thailand cultivars. Notably, the silkworm ash content is typically reported to range from 3% to 10%, with the current findings falling within the upper quartile of this range [30]. In terms of fat content, silkworms fed on Ex-Thika mulberry exhibited the highest levels, suggesting a positive correlation with the nutritional quality of the plant. A similar pattern was observed for crude protein, with Noi-EX-Thailand mulberry accruing the highest protein levels. For carbohydrates, silkworms fed on Victory1 mulberry cultivar showed the highest content. We reported nutritional values that fall within the highest range compared to those documented in the literature. Specifically, the reported values for crude fat, crude protein, and carbohydrates range from 30–50%, 45–60%, and 0.78–6.57%, respectively [31, 32]. Feeding silkworms with different mulberry cultivars significantly enhanced the nutritional quality of the silkworm pupae, although the observed values remained within the upper quartile of previously reported ranges. Future research should explore innovative strategies to further optimize the nutritional profile of mulberry leaves, with the aim of elevating the quality of silkworm pupae beyond current benchmarks.

As shown in Table 2, the amino acid content in silkworm pupae was significantly higher compared to that in the mulberry leaves. This increase may be attributed to the insects’ ability to ingest compounds from a plant-based diet and subsequently metabolize and concentrate them into novel derivatives, which could offer enhanced nutritional and therapeutic benefits [33]. The essential amino acids identified in this study align with findings from previous research [4, 9, 31, 32]. All essential amino acids were quantified in both the plants and insects, except for tryptophan, which may have been degraded during acid hydrolysis. Future studies are recommended to assess this critical amino acid more accurately [34].

The mineral composition of silkworms reared on the various mulberry cultivars are consistent with previously reported findings [29–31, 35]. However, the *Noi-EX-Thailand* cultivar significantly enhanced key minerals such as zinc, calcium, and magnesium. This underscores the importance of selecting the appropriate mulberry variety to optimize the nutritional content of silkworms. Further research is needed to investigate why rearing silkworm on *Noi-EX-Thailand* resulted in elevated zinc and potassium levels, and whether environmental or genetic factors contributed to these enhanced mineral concentrations.

The vitamin data were impressive, as all cultivars exhibited elevated levels of vitamin C, particularly *Victory1* and *Noi-EX-Thailand*. This indicates that silkworms fed these vitamin-rich leaves benefited from enhanced overall nutritional quality. Thiamine levels in silkworm pupae remained consistent across cultivars, suggesting a strong ability to retain this vitamin regardless of dietary differences. In contrast, riboflavin content showed minimal variation, indicating that silkworms can effectively synthesize or retain this vitamin during development. Notably, niacin levels were higher in pupae fed *Ex-Thika* leaves, reflecting the leaf’s nutrient profile and promoting better retention. Additionally, *Noi-EX-Thailand* contributed to increased pyridoxine retention, while *Ex-Thika* also enhanced folic acid levels, highlighting its benefits for improving the nutritional profile of silkworms. Overall, these findings underscore the importance of selecting appropriate mulberry cultivars to optimize vitamin composition, thereby enhancing nutrient retention in silkworms and potentially improving growth, development, and silk quality. Vitamins are crucial for various metabolic processes, including tissue growth, energy metabolism, protein metabolism, and blood cell production, emphasizing their significance in the diets of both silkworms and other animals [20, 36, 37].

The correlation between the nutrient composition of mulberry leaves and the quality of silkworm pupae aligns with findings from [37–39], which emphasize that silkworms feed exclusively during their larval stage. The growth and development of these larvae are heavily influenced by the nutritional quality of the mulberry leaves they consume. Consequently, the nutritional background during the larval stage significantly impacts the subsequent status of the larvae, pupae, adults, and cocoons. Furthermore, silkworm pupae can serve as a valuable energy source, supported by a strong correlation observed in this study between the Nitrogen Free Extract (NFE) from mulberry leaves and that from silkworm pupae (r = 0.6847, P<0.05) (Table 5). Silkworm pupae, typically regarded as a byproduct of the silk industry, can serve as an unconventional, cost-effective protein and energy source for poultry feed. Research indicates they can effectively replace fish meal or soybean meal in broiler diets, offering an affordable alternative [40]. Additionally [41], highlighted that feeding dried silkworm pupae to poultry improved growth rates and egg quality in hens, as well as enhancing survival rates, feed conversion rates, and specific growth rates in fish. Silkworm pupae are recognized as a high-quality protein and an important source of nutrients [35, 36, 40, 41]. The significance of mineral content in mulberry plant material is further emphasized by [42] who indicated that high ash content reflects a substantial presence of inorganic nutrients in the succeeding silkworm pupae. Moreover [4] pointed out that mulberry leaves play a crucial role in determining the mineral content of silkworms, largely due to the bioaccumulation of minerals through nutrition.

The variability in mineral content of silkworm pupae fed different mulberry leaf cultivars may be linked to mineral availability in the soil where the mulberry is cultivated. However, factors such as soil contamination by heavy metals [43], the specific mulberry cultivar, and the timing of the harvest also significantly influence mineral accumulation [31]. This study’s findings suggest that silkworm pupae could be an excellent source of minerals, consistent with earlier reports [32, 44].

Silkworm pupae stand out as an exceptional high-quality nutrient source when compared to other insects widely used in food and feed [Table 9]. While insects like crickets and mealworms offer comparable or slightly higher levels of protein [27, 45–48], silkworm pupae are particularly richer in essential cereal limiting amino acids [lysine and methionine] as well as threonine and leucine. Besides protein, silkworm pupae also provide a variety of vitamin B, A and E.

**Table 9:** Comparative nutritional profile of silkworm pupae vs. other insect species for food and feed applications.

Insect species	Crude protein	Key amino acids	Vitamins	Comparison to *B*. *mori*	Reference
Silkworm pupae	~50–60%	High in lysine, methionine, threonine, and leucine	Vitamin B2, B5, and some A and E	Baseline for comparison	[2, 20]
Black soldier fly larvae	~40–45%	High in essential amino acids like leucine, lysine, and valine	Vitamin B12, B2, and minerals like calcium and magnesium	Lower in protein content than *B*. *mori*, high in essential amino acids	[49, 50]
Yellow mealworm	~50–60%	Rich in essential amino acids (lysine, methionine)	Vitamin B1, B2, B3, and B5	Similar protein content, good vitamin profile	[45]
Housefly larvae	~40–50%	Good source of arginine, leucine, and lysine	B-vitamins (B1, B2, B3), and some vitamin E	Lower in protein content, good amino acid profile	[51–53]
Cricket	~60–70%	Complete amino acid profile, rich in leucine, lysine, and threonine	Vitamin B12, B2, B3, B5, folate	Higher protein content than *B*. *mori*, complete amino acid profile	[27, 47, 48]
Ants	~40–50%	Rich in glutamine, leucine, and lysine	Vitamin A, B-vitamins (B1, B2, B12)	Lower protein content than *B*. *mori*, good vitamin profile	[54, 55]
Termites	~35–45%	High in essential amino acids like arginine, valine	Vitamin B1, B2, and some A	Lower protein content than *B*. *mori*, similar amino acid profile	[56–58]

Some of the shortcomings of the current studies included, limited number of mulberry cultivars, not all critical nutritional data [e.g., fiber, total caloric values, bioactive compounds etc.] were generated, and safety quality of the pupae was not investigated. All these aspects are new areas of research that have been recommended from this study as to make informed decision on their development as a viable nutrient-rich source for animal nutrition and health. This study opens new doors for optimizing silkworm pupae production for food, feed and pharmaceutical applications. By selecting the most suitable mulberry cultivars, farmers can enhance the nutritional value of silkworm pupae, contributing to more sustainable and excellent food production systems, offering an innovative solution to global nutrition and food security challenges.

## 5. Conclusions

This study highlights the nutritional benefits of silkworm (*Bombyx mori*) pupae, identifying them as rich sources of protein, essential amino acids, vitamins, and minerals. Specifically, the Noi-EX-Thailand and Ex-Thika mulberry cultivars markedly improved the pupae’s crude protein (57.9%) and fat (44.3%) content, while the Victory1 and Noi-EX-Thailand cultivars enhanced vitamin C and potassium levels. Our findings highlight a strong correlation between the nutrient composition of mulberry leaves and the quality of silkworm pupae, emphasizing the necessity of selecting suitable mulberry cultivars for optimal silkworm rearing. Overall, the findings advocate for the careful selection of mulberry cultivars to improve larval development, and the nutritional quality of pupae, reinforcing their potential as sustainable food and feed sources. Future research should further explore these implications to enhance sericulture practices. It should also prioritize safety considerations, which are crucial when evaluating any potential food or feed source. Additionally, investigating the impact of processing methods on the nutritional profile of silkworm pupae, especially as a novel ingredient, is essential for ensuring their suitability for human and animal consumption.
